# Desialylated Mesenchymal Stem Cells-Derived Extracellular Vesicles Loaded with Doxorubicin for Targeted Inhibition of Hepatocellular Carcinoma

**DOI:** 10.3390/cells11172642

**Published:** 2022-08-25

**Authors:** Chunyan Yang, Zixuan Guan, Xincheng Pang, Zengqi Tan, Xiaomin Yang, Xiang Li, Feng Guan

**Affiliations:** 1 Key Laboratory of Resource Biology and Biotechnology in Western China, Ministry of Education, Provincial Key Laboratory of Biotechnology, College of Life Sciences, Northwest University, Xi’an 710069, China; 2 Institute of Hematology, School of Medicine, Northwest University, Xi’an 710069, China; 3 Department of Breast Surgery, The First Affiliated Hospital of Xi’an Jiaotong University, Xi’an 710069, China; 4 Department of Breast Surgery, Tumor Hospital of Shaanxi Province, Xi’an 710069, China

**Keywords:** extracellular vesicles, drug delivery, desialylation, ASGPR, liver targeting

## Abstract

Hepatocellular carcinoma (HCC) is one of the dominating causes of cancer-related death throughout the world. Treatment options for patients with HCC vary, however, the lack of effective targeted drugs is the major reason for death in advanced HCC patients. In this study, a delivery system based on mesenchymal stem cell (MSC)-derived extracellular vesicles (EVs) loaded with doxorubicin (Dox) was developed. In this system, we initially erased terminal linked α2–3 and α2–6 sialic acids on the surface of EVs by neuraminidase. The exhibition of galactose (Gal) and N-acetylgalactosamine (GalNAc) residues in treated MSC-EVs can specifically be recognized by asialoglycoprotein receptor (ASGPR) of hepatoma cells. Compared to free Dox and Dox-loaded EVs, desialylated EVs loaded with Dox significantly presented the improved cellular uptake, prioritized targeting efficacy, and had a better inhibiting effect in vitro and in vivo. Overall, the results of the present study of the demonstrated delivery system using desialylated MSC-EVs suggest its therapeutic potential for HCC.

## 1. Introduction

Primary liver cancer is the third most deadly malignant tumor in the world, of which hepatocellular carcinoma (HCC) accounts for 75–85% [1]. Despite advances in HCC administration, medical therapy still exhibits limited benefit on patient survival [2]. The most commonly used doxorubicin (Dox) is hydrophilic molecules that suffers from the drawbacks of rapid clearance, a short circulation time and off-target effect [3]. Herein, there is a pressing need to explore more effective and safer treatments for HCC.

Mesenchymal stem cells (MSCs) are the most extensively used stem cells in the regenerative medicine field. MSC-derived extracellular vesicles (EVs) are nanoparticles secreted by MSCs that collectively exhibit typical exosome-associated proteins and carry a rich diversity of RNAs/proteins [4]. MSC-EVs illustrate the features of natural biocompatibility, inherent long-circulation ability in vivo, limited immunogenicity, low cytotoxicity, easy passing through physiological barriers, and potential specific targeting properties [5]. Additionally, MSC-EVs present bioactivity by delivering the therapeutic cargo of RNAs and proteins to the recipient cells [6]. Therefore, MSC-EVs are increasingly used as an intermediary for many MSC-associated therapeutic potencies.

As one of the most common post-translational modifications, glycosylation can be found on the surface of EVs and play functional roles. For instance, a low level of the bisecting N-acetylglucosamine (GlcNAc) structure was observed in EVs from high-metastatic breast cancer cells, while enhanced bisecting GlcNAc structures on EVs inhibited their carcinogenic effects [7]. Galectin-5, one member of the family of β-galactoside binding proteins, expressed on the surface of rat reticulocyte exosomes modulated its uptake by macrophages [8]. Sialic acids on exosomes from human adipose-derived mesenchymal stem cells were related to sialic acid-binding immunoglobulin-like lectin (siglec)-mediated cellular uptake [9]. Sialic acid modification, a very common glycosylation in mammalian cells, is located at the terminal of glycans and plays an important role in cell recognition and interaction [10]. Desialylation of EVs derived from mouse liver cells could affect recipient endocytosis by different human cells [11] and alter their distribution in mice [12]. This may provide a clue to use the desialylated EVs as a specific receptor-mediated drug delivery system.

The asialoglycoprotein receptor (ASGPR), mainly distributed on the surface of hepatocytes but rarely expressed in other cells, has drawn great attention to the study of receptor-mediated liver targeting [13]. ASGPR has a high affinity for a variety of carbohydrates, especially galactose (Gal) and N-acetylgalactosamine (GalNAc) [14]. Gal or GalNAc or their derivatives decorated on different nanoparticles could achieve more efficient drug delivery in HCC. For example, Gal and GalNAc were coupled with cholesterol to produce the galactosylated lipids, which showed greater accumulation in the liver than unmodified liposomes [15]. In our preliminary data, we found enriched sialic acids on surface of MSC-EVs. Herein, we proposed that the removal of sialic acids on MSC-EVs to expose Gal or GalNAc may facilitate the ASGPR-mediated targeting of the HCC cells.

In this study, sialic acids on MSC-EVs were removed by neuraminidase and a desialylated MSC-EV based nanoparticle system composed of chemotherapeutic Dox was established. The physicochemical properties and antitumor activity in vitro and in vivo of this nanoparticle were evaluated.

## 2. Materials and Methods

### 2.1. Cell Culture

The human umbilical mesenchymal stem cells were received from the Department of Hematology, Shaanxi Provincial People’s Hospital. Human urothelial cells HCV-29 were gifted by Dr Sen-itiroh Hakomori (The Biomembrane Institute, Seattle, WA, USA). Bone marrow-derived stromal cell line HS5 and HS27a were gifts from Professor H. Joachim Deeg (Fred-Hutchinson Cancer Research Center, Seattle, WA, USA). Human liver cancer cell line HepG2, human liver normal cell line HL-7702, and human gastric epithelial cell line GES-1 were purchased from the Cell Bank of the Chinese Academy of Sciences (Shanghai, China). HepG2, GES-1, HS5, and HS27a cells were cultured in a DMEM medium with high glucose (Biological Industries, Beit Haemek, Israel), while HL-7702 and HCV-29 cells were cultured in RPMI 1640 medium (Biological Industries) at 37 °C in 5% CO_2_. All mediums were supplemented with 10% fetal bovine serum (FBS, Biological Industries) and 1% of penicillin/streptomycin (HyClone, Provo, UT, USA).

### 2.2. Isolation of MSC-EVs

MSC-EVs were purified from the FBS-free medium of MSCs by differential ultracentrifugation in the following steps. First, the conditioned medium was centrifuged at 500× *g* for 10 min and 2000× *g* for 20 min at 4 °C. The supernatant was centrifuged at 11,000× *g* for 30 min at 4 °C. Next, the pellet was harvested by ultracentrifugation at 110,000× *g* for 70 min (Optima XE-100 ultracentrifuge, Beckman coulter life Sciences, Indianapolis, IN, USA). The supernatant was removed and the pellet was resuspended in PBS, followed by another 70 min ultracentrifugation at 110,000× *g* at 4 °C. The final pellet was resuspended in PBS and stored at −80 °C. The protein concentration of the purified MSC-EVs was quantified by the BCA Protein Assay Kit (Beyotime Institute of Biotechnology, Haimen, China).

### 2.3. Characterization of MSC-EVs

The size distribution and zeta potential of the EVs were analyzed by dynamic light scattering (DLS) using Nano-ZS ZEN3600 (Malvern, UK).

For morphology observation, 30 μL of the purified EVs were dropped on the carbon film covered copper grids, dried for 5 min, stained with 2% uranyl acetate for 1 min, and observed under transmission electron microscopy (TEM, H-7650, Hitachi, Tokyo, Japan) at 80 kV.

For biomarker analysis, EV positive and negative markers were examined by Western blotting using the following antibodies: CD63 (1:1000, ab134045), TSG101 (1:1000, ab83, Abcam, Cambridge, MA, USA)), CD81 (1:1000, sc-23962, Santa Cruz Biotechnology, Santa Cruz, CA, USA), Alix (1:1000, 2171S), and Calnexin (1:1000, 2679S, Cell Signaling Technology, Beverly, MA, USA). A total of 30 μg EV samples were boiled at 95 °C for 10 min, separated by 10% SDS-PAGE and transferred onto polyvinylidene difluoride (PVDF) membranes (Bio-Rad, Hercules, CA, USA). Membranes were blocked with 5% (*w*/*v*) non-fat milk in TBST at room temperature (RT) for 1 h, incubated overnight with the primary antibodies above, followed by incubation with HRP-conjugated secondary antibodies (1:5000, Beyotime). Bands were visualized by enhanced chemiluminescence (ECL, Vazyme Biotech, Nanjing, China).

### 2.4. Doxorubicin Loading into MSC-EVs

Purified MSC-EVs were normalized to a protein concentration of 1 mg/mL for the following studies. The chemotherapeutic drug doxorubicin hydrochloride (Dox, MedChemExpress, Monmouth Junction, NJ, USA) at 500 μg/mL was added to an equal volume of MSC-EVs. After loading the drug with different methods, unencapsulated Dox was removed by ultrafiltration using Amicon ultra centrifugal filters (10 kDa, Millipore, Billerica, MA, USA) at 14,000× *g* for 15 min. The concentration of Dox in PBS was quantified by measuring the optical density at 480 nm on a microplate reader (Synergy 2, BioTek, Winooski, VT, USA) [16]. The drug encapsulation efficiency (EE%) was calculated by dividing the amount of loaded drug by that of the total drug. The drug loading efficiency (DL%) was calculated by dividing the amount of loaded drug by that of the drug-loaded EVs.

The reaction conditions are summarized in Supplementary Table S1 and described as follows. The incubation method was performed by mixing EVs and Dox evenly and incubation at 37 °C for 30 min. The ultrasonic method was performed by the following steps: 10 s sonicate, 10 s pause, and repeating three times by an ultrasonic cell disruptor (TL-1000Y, Tenlin, Yancheng, China). Electroporation was conducted with the condition of a 1 ms pulse at a 150 V voltage, repeating this five times by an electroporation instrument (Gene Pulser Xcell, Bio-Rad). Afterward, the mixture was incubated at 37 °C for 30 min to ensure that the EV membrane fully recovered. Freeze–thaw cycles were performed by freezing at −80 °C for 30 min and thawing at RT. The freeze–thaw cycles were repeated three times.

The particle size and morphology of the drug-loaded EVs were further determined via DLS and TEM, as described above.

### 2.5. Doxorubicin Release and Retention

The doxorubicin release from EVs was evaluated by ultrafiltration. Dox-loaded MSC-EVs (E-Dox) or an equal amount of Dox were added to the inner ultrafiltration tube (10 kDa, Millipore), and PBS was added to the outer collection tube. The concentration of Dox in the inner tube was quantified spectrophotometrically at 480 nm at 12, 24, 36, 48, 60, and 72 h, respectively. The percentage of drug release at each time point was recorded and calculated.

The intracellular drug retention was performed as follows. HepG2 cells (1 × 10^5^ cells) were seeded in 12-well plates and treated with Dox and E-Dox for 12 h. The cells were washed with PBS and detached with trypsin at 0, 2, 4, 6, 12, and 24 h. The intracellular residual Dox was detected by flow cytometry (ACEA Biosciences, San Diego, CA, USA) using a 530 nm LP filter, and the corresponding drug retention rate was calculated.

### 2.6. Desialylation of EVs

EVs or E-Dox (30 μg protein) were incubated with different amounts (0, 0.5, 1, 2, 4, 6, 8 U) neuraminidase (Neu, S31133, Yuanye, Shanghai, China) at 37 °C for 30 min, and excess neuraminidase was removed by ultracentrifugation. The removal of sialic acid modification on EVs was detected by lectin blotting. Briefly, the EV samples were separated by 10% SDS-PAGE, transferred onto PVDF membranes, blocked in 3% BSA (*w*/*v*) in TBST, and incubated overnight with biotinylated lectins (2 μg/mL, Vector Labs, Burlingame, CA, USA) including *Sambucus Nigra* Lectin (SNA, B-1305), *Maackia Amurensis* Lectin II (MAL II, B-1265), *Peanut Agglutinin* (PNA, B-1075), *Erythrina Cristagalli* Lectin (ECL, B-1145), *Lens Culinaris Agglutinin* (LCA, B-1045-5), *Phaseolus Vulgaris Leucoagglutinin* (PHA-L, B-1115-2), and succinylated *Wheat Germ Agglutinin* (WGA, B-1025S-5). Membranes were incubated with ABC reagent (Vectastain Elite ABC peroxidase Kit, Vector Labs) for 1 h at RT according to the manufacturer’s instructions, and bands were visualized by ECL as above.

### 2.7. MSC-EV Uptake

MSC-EVs were labeled with ExoTracker [17], and incubated with recipient HepG2 cells for 1 h. The uptake of MSC-EVs was analyzed by confocal laser scanning microscopy (TCS SP8, Leica Microsystems, Wetzlar, Germany) or flow cytometry (ACEA) using a 675 nm LP filter.

Free Dox, E-Dox, or desialylated Dox-loaded MSC-EVs (dsE-Dox) were incubated with HepG2 cells for 12 h, respectively. Cells were subsequently washed three times with PBS, trypsinized, and suspended in 300 μL PBS. The autofluorescence of Dox was detected by flow cytometry (ACEA).

### 2.8. ASGPR-Mediated Targeting Studies

HepG2 cells (1 × 10^5^ cells per well) were seeded in 12-well plates overnight. Different concentrations of D-galactose (Gal, Sigma-Aldrich, St. Louis, MO, USA) and N-acetyl-D-galactosamine (GalNAc, Aladdin, Shanghai, China) were added to culture medium for different times. The cells were treated with PBS, Dox, E-Dox, and dsE-Dox in the FBS-free medium, respectively. After incubating for 12 h, cells were washed three times with PBS, detached with trypsin, suspended in 300 μL PBS, and the autofluorescence of Dox was analyzed by flow cytometry (ACEA).

Recipient cells including HepG2, HL-7702, GES-1, HCV-29, HS5, and HS27a (1 × 10^5^ cells per well) were individually seeded in 12-well plates and cultured overnight. E-Dox and dsE-Dox were incubated with recipient cells for 12 h, respectively, and the uptake was detected by using flow cytometry, as described above.

### 2.9. Cell Proliferation

HepG2 cells (1 × 10^4^ cells per well) were seeded in 96-well plates for 24 h and treated with free Dox, E-Dox, dsE-Dox, or untreated MSC-EVs for different times at 37 °C. Cell proliferation was performed with the CCK-8 assay (Beyotime) according to the manufacturer’s instructions.

### 2.10. Cell Apoptosis

For apoptosis analysis, HepG2 cells (2 × 10^5^ cells per well) were seeded in 6-well plates. After overnight culture, cells were treated with PBS, free Dox, E-Dox, and dsE-Dox (2 μmol/L Dox) for 24 h and stained with Annexin V-APC/7-AAD Kit (BioLegend, San Diego, CA, USA), according to the manufacturer’s instructions. The apoptosis rate was measured by flow cytometry (ACEA) and analyzed by FlowJo software (TreeStar Inc., Ashland, OR, USA).

### 2.11. Transwell Assay

HepG2 cells (2 × 10^4^) were seeded in the upper chamber of the cell culture inserts (diameter 24 mm, pore size 8 μm, Corning, NY, USA) and cultured in the FBS-free medium. Complete medium was added to the bottom chamber. After 24 h incubation, cells that migrated across the membrane were stained with 0.1% crystal violet and imaged with a microscope.

### 2.12. In Vivo Antitumor Efficacy

The xenograft tumor model was generated by subcutaneously injection of 1 × 10^7^ HepG2 cells into the right side of the fossa axillaries of Balb/c nude mice (five weeks old). When the tumor volume was about 120 mm^3^, mice were randomly divided into four groups. Mice were intravenously injected with 100 μL of PBS, Dox, E-Dox, or dsE-Dox (2 mg Dox/kg) every three days, six times. The tumor volume and body weight were recorded. The tumor size was calculated according to the formula: tumor volume (mm^3^) = 0.5 × length × width^2^. At the end of the experiments, the mice were humanely sacrificed to dissect, and the tumors were harvested, weighed, and evaluated by Ki67 immunohistochemistry. The heart, liver, spleen, lung, and kidney were stained with hematoxylin and eosin (H&E). All animal experiments were approved by the Institutional Animal Care and Use Committee of Northwest University.

### 2.13. Statistical Analysis

Data were presented as the mean values ± SD. All statistical analysis was performed using GraphPad Prism 7 (GraphPad Software, San Diego, CA, USA). The statistical significance of differences between the means of two groups was evaluated by the unpaired *t*-test and two-way ANOVA. Differences with *p* < 0.05 were considered statistically significant. The significant levels were shown as * *p* < 0.05, ** *p* < 0.01, *** *p* < 0.001.

## 3. Results

### 3.1. Isolation and Characterization of MSC-EVs

**Figure 1:**
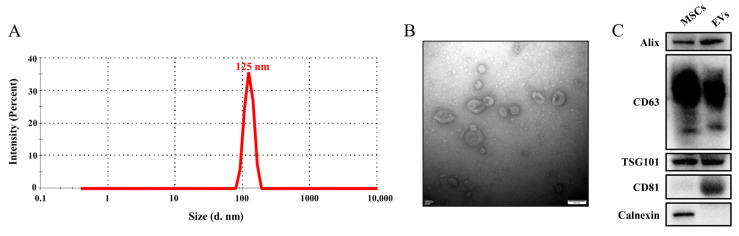
Characterization of the MSC-EVs. (**A**) The size distributions of the MSC-EVs based on intensity by the DLS measurements. (**B**) Representative TEM images of the MSC-EVs. Scale bar, 100 nm. (**C**) Western blotting analysis of the EV markers.

EVs were purified from the culture supernatants of MSCs by ultracentrifugation. The size and morphology of the MSC-EVs were assessed using DLS and TEM. EVs showed a narrow size distribution with a peak value at 125 nm (Figure 1A). The MSC-EVs presented a symbolic saucer-like bilayer membrane structure (Figure 1B). The typical markers of EVs such as Alix, TSG101, CD63, and CD81 were confirmed by Western blotting, while calnexin, which existed only in cells, was not detected in the EVs (Figure 1C). These characteristics are consistent with the MISEV 2018 guidelines [18].

### 3.2. In Vitro Cytotoxicity Study of Drug-Loaded MSC-EVs

Dox was encapsulated into purified MSC-EVs to form a delivery system. To optimize the loading efficiency, we explored four different methods including incubation, ultrasound, electroporation, and freeze–thaw cycles. Our data suggested that the ultrasonic strategy obtained the highest encapsulation efficiency (EE, 34.20%) and drug loading efficiency (DL, 14.60%) (Table S1). Therefore, ultrasonic strategy was selected in the following study. The size and morphology of the EVs and drug loaded EVs displayed no difference in the DLS and TEM analysis (Figure S1a,b).

**Figure 2:**
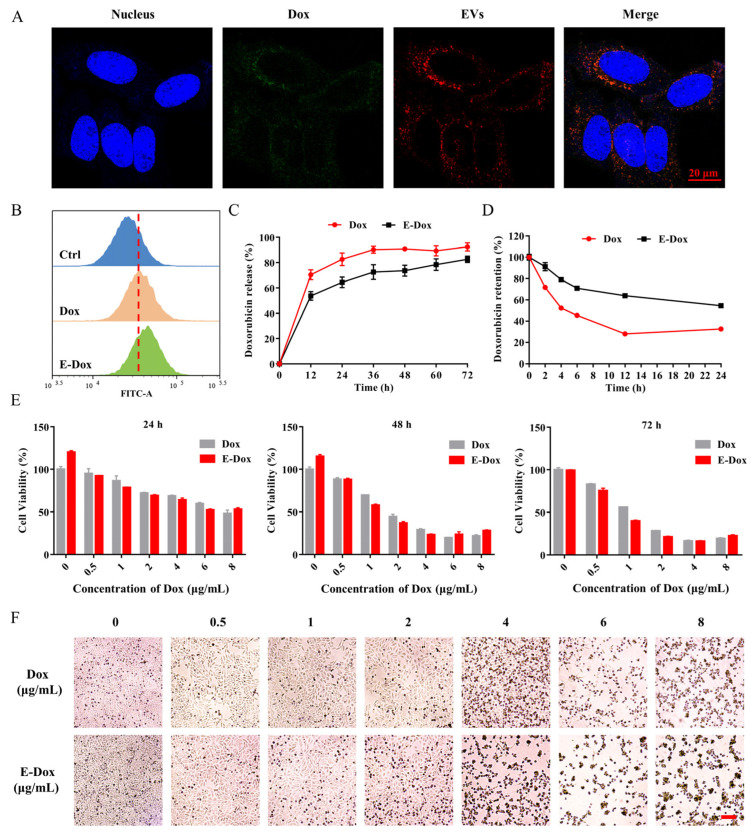
Cellular uptake, drug release, and cytotoxicity of E-Dox. (**A**) HepG2 cells were incubated with E-Dox for 1 h and photographed under confocal microscopy. Blue, Hoechst 33,258 for nucleus. Red, ExoTracker for labeled EVs. Green, autofluorescence of Dox. Scale bar, 20 μm. (**B**) Uptake of Dox or E-Dox (loaded with 2 μmol/L Dox) by the HepG2 cells were detected by measuring the autofluorescence of Dox using flow cytometry. (**C**) The time dependent drug release of Dox and E-Dox. (**D**) The retention rate of Dox and E-Dox in the HepG2 cells were detected by flow cytometry. (**E**) The HepG2 cells were treated with free Dox or E-Dox at a dose dependent manner for 24, 48, or 72 h. The relative cell viability was measured by the CCK-8 assay. (**F**) The cell viability of HepG2 after treatment with Dox or E-Dox for 24 h. Scale bar, 100 μm.

Next, the drug loaded EVs were labeled with ExoTracker, a novel exosome labeling fluorescent probe, and fed to hepatoma cell HepG2. The co-localized fluorescence signals from Dox itself and ExoTracker indicated that E-Dox was successfully engulfed into HepG2 cells (Figure 2A). The fluorescent signal of free Dox was also observed in the HepG2 cells (Figure S2). The endocytosis of E-Dox into recipient cells was detected by flow cytometry (Figure 2B and Figure S4). To test the toxic effects of E-Dox, drug release and cytotoxicity assays were performed. The process of drug release from the EVs showed a much slower release rate of E-Dox than free Dox (Figure 2C). The retention ratio of the E-Dox in cells was higher than that of free Dox (Figure 2D). The recipient HepG2 cells after treatment with E-Dox or free Dox showed similar cytotoxic outputs (Figure 2E,F).

### 3.3. Desialylated MSC-EVs Targeting ASGPR on Hepatic Cells

**Figure 3:**
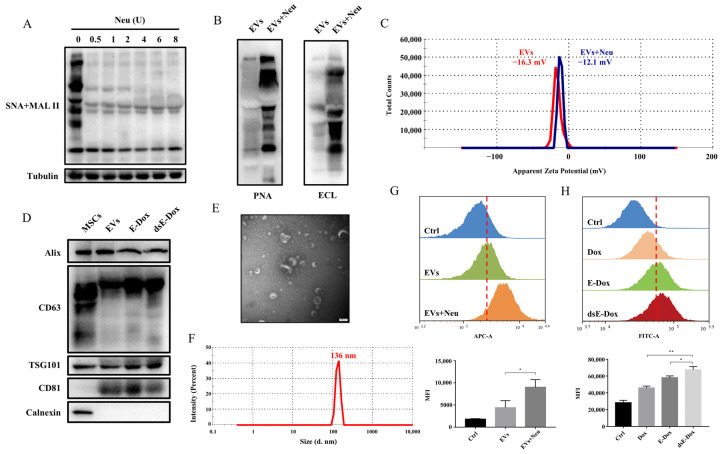
The effect of desialylation on the cellular uptake of MSC-EVs. (**A**) MSC-EVs were treated with different concentrations of neuraminidase for 30 min at 37 °C. The sialic acid level was determined by lectin blotting. (**B**) The Gal and GalNAc levels of EVs were detected by lectin blotting with 4 U neuraminidase treatment at 37 °C for 30 min. (**C**) The zeta potential of EVs treated with or without neuraminidase was estimated by DLS. (**D**) Positive markers, Alix, CD63, TSG101, and CD81, and negative marker calnexin of the MSC-EVs, E-Dox and dsE-Dox were analyzed by Western blotting. (**E**,**F**) The morphology and size of the desialylated MSC-EVs were detected by TEM and DLS, respectively. Scale bar, 100 nm. (**G**) MSC-EVs and desialylated MSC-EVs were labeled with ExoTracker, and their cellular uptake were analyzed by flow cytometry. (**H**) Uptake of free Dox, E-Dox, and dsE-Dox (loaded with 2 μmol/L Dox) were analyzed by measuring the autofluorescence of Dox using flow cytometry. The data were presented as the mean values ± SD. * *p* < 0.05, ** *p* < 0.01.

Consistent with another study [19], sialylation was also found in the MSC-EVs, revealed by lectins MAL II and SNA, which recognize the α2–3 and α2–6 sialic acids, respectively (Figure 3A). To further investigate whether the changes in surface glycosylation could affect the targeting of EVs, neuraminidase was used to remove sialic acids on the MSC-EVs. Our results confirmed the α2–3 and α2–6 sialic acid residues on the surface of the EVs were removed and terminal Gal and GalNAc were significantly exposed after neuraminidase treatment with the optimal amount of 4 U (Figure 3A,B and Figure S3a). To exclude other carbohydrate-related enzyme activities of neuraminidase, lectin blotting with more lectins was performed, which demonstrated that levels of fucose recognized by LCA, β1–6 branched GlcNAc recognized by PHA-L, and GlcNAc residue recognized by WGA were not changed in the neuraminidase treated EVs (Figure S3b). Due to the negative charge of the sialic acids, the zeta potential of EVs was increased to −12.1 mV from −16.3 mV after neuraminidase treatment. The dispersion stability of EVs after the removal of sialic acid did not significantly change (Figure 3C) and the typical EV markers appeared on the dsE-Dox compared to the untreated EVs and E-Dox (Figure 3D). Additionally, the desialylated EVs exhibited a similar morphology and size as the non-treated EVs (Figure 3E,F). Furthermore, neuraminidase treatment significantly promoted the cellular uptake of MSC-EVs and E-Dox (Figure 3G,H). These data revealed that the removal of sialic acids exposed more Gal and GalNAc on the MSC-EVs and facilitated their endocytosis.

**Figure 4:**
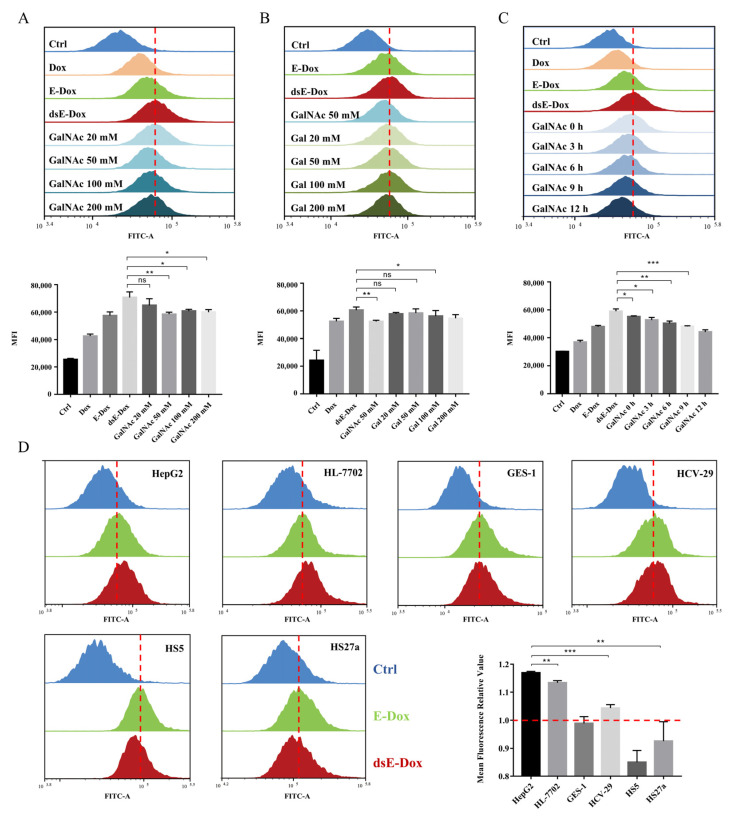
The targeting capability of MSC-EVs in vitro. (**A**,**B**) HepG2 cells were pretreated with various concentrations of GalNAc or Gal for 4 h, and the cellular uptake of dsE-Dox (loaded with 2 μmol/L Dox) was detected by flow cytometry. (**C**) HepG2 cells were pretreated with 50 mM GalNAc for 0, 3, 6, 9, 12 h, and the cellular uptake of dsE-Dox (loaded with 2 μmol/L Dox) was detected by flow cytometry. (**D**) The uptake of dsE-Dox (loaded with 2 μmol/L Dox) by HepG2, HL-7702, CES-1, HCV-29, HS5, and HS27a cells were assayed by flow cytometry. The mean fluorescence relative value refers to the MFI ratio of dsE-Dox and E-Dox. The data were presented as the mean values ± SD. * *p* < 0.05, ** *p* < 0.01, *** *p* < 0.001.

Our hypothesis is that exposure of the Gal/GalNAc residues after the removal of sialic acids on MSC-EVs enhances the binding affinity with ASGPR on hepatoma cells. To prove this, we added different concentrations of Gal and GalNAc to block the ASGPR in advance. The data showed that the endocytosis of dsE-Dox by HepG2 cells was distinctly inhibited after pre-incubation with GalNAc (Figure 4A). However, compared with GalNAc, the inhibitory effect of Gal on cellular uptake was not remarkable (Figure 4B). With a longer incubation of GalNAc, the endocytosis of dsE-Dox was more inhibited (Figure 4C).

Furthermore, we used five other cell lines including the hepatocyte cell HL-7702, gastric epithelial cell GES-1, urothelial cell HCV-29, and bone marrow stroma cells HS5 and HS27a to validate the above results. Only HL-7702 cells showed an increased cellular uptake for dsE-Dox. The GES-1, HCV-29, HS5, and HS27a cells showed no difference, and even a slightly decreased cellular uptake (Figure 4D). This may imply that increased EV uptake was related to the ASGPR-mediated endocytosis. Moreover, in comparison with HL-7702, the viability of HepG2 was significantly inhibited by dsE-Dox or free Dox (Figure S5a,b).

In general, our data suggest that the removal of sialic acids on MSC-EVs can exhibit more Gal and GalNAc residues, and thus facilitate the binding with ASGPR on hepatoma cells.

### 3.4. Inhibitory Effect of Desialylated MSC-EVs on HCC

**Figure 5:**
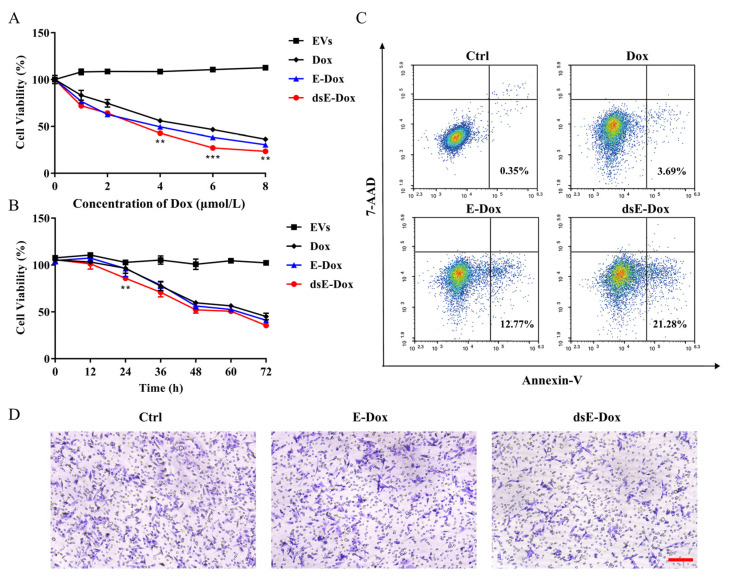
The in vitro antitumor activity of dsE-Dox. (**A**) The viability of HepG2 cells treated with Dox, E-Dox, dsE-Dox (loaded with 0, 2, 4, 6, 8 μmol/L Dox), or free EVs for 48 h was detected by the CCK-8 assay. (**B**) The effects of Dox, E-Dox, dsE-Dox (loaded with 4 μmol/L Dox), and free EVs (500 μg/mL protein concentration) on HepG2 cell viability at different time points were detected by the CCK-8 assay. (**C**) HepG2 cells were treated with PBS, Dox, E-Dox, or dsE-Dox (loaded with 2 μmol/L Dox) for 24 h and stained with Annexin V-APC/7-AAD. Cell apoptosis was measured by flow cytometry. (**D**) HepG2 cells were treated with PBS, E-Dox, and dsE-Dox (loaded with 2 μmol/L Dox) for 24 h and the migration ability was detected by the Transwell assay. Scale bar, 150 μm. The data were presented as the mean values ± SD. ** *p* < 0.01, *** *p* < 0.001.

Encouraged by the above data, we explored whether dsE-Dox could enhance an inhibitory effect on HCC. Compared to other groups, the viability of HepG2 treated with dsE-Dox was significantly inhibited at both dose- and time-dependent manners (Figure 5A,B). The proportion of apoptotic cells in the dsE-Dox treated group was significantly higher than in the other groups (Figure 5C). The dsE-Dox also showed a significantly inhibitory effect on cell migration, confirmed by the Transwell assay (Figure 5D). Collectively, these data suggest that the desialylated MSC-EV loading system could efficiently deliver Dox into recipient HepG2 to inhibit its proliferation and migration, and promote its apoptosis.

### 3.5. Targeted Therapy of Desialylated MSC-EVs on HCC In Vivo

**Figure 6:**
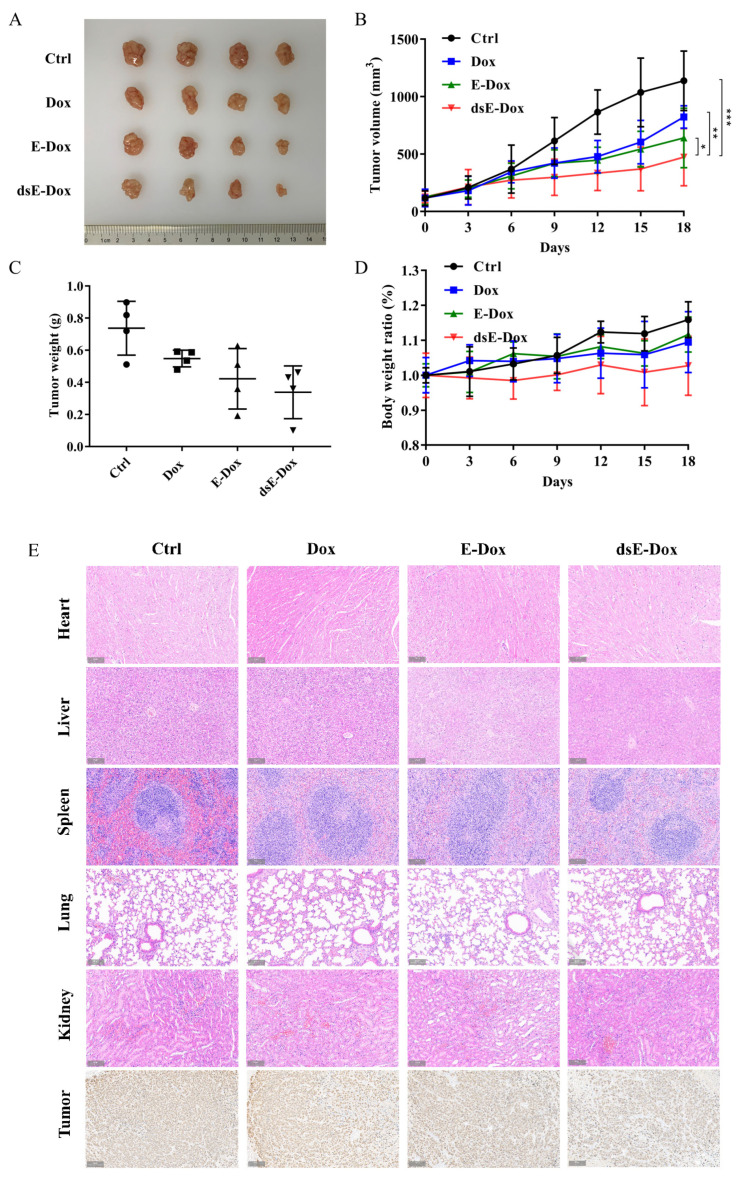
The in vivo antitumor activity of dsE-Dox. (**A**) HepG2 tumor-bearing mice treated with PBS, Dox, E-Dox, or dsE-Dox (equal to 2 mg/kg Dox) every 3 days via the tail vein. The tumor was isolated and photographed. (**B**) The tumor growth curves (n = 4, mean ± SD). (**C**) The weight of the excised tumor. (**D**) The body weight ratio. (**E**) H&E staining of the heart, liver, spleen, lungs, and kidney. Ki67 staining of the tumor tissues. Scale bar, 100 μm. The data were presented as the mean values ± SD. * *p* < 0.05, ** *p* < 0.01, *** *p* < 0.001.

To investigate the targeting effect of desialylated MSC-EVs in vivo, we constructed a xenograft mouse model by subcutaneously injecting HepG2 cells into the Balb/c nude mouse. The results showed that the size, volume, and weight of the tumor in the dsE-Dox treated group were significantly suppressed (Figure 6A–C), while the body weight of the nude mice did not change obviously (Figure 6D). Ki67 staining showed that dsE-Dox significantly inhibited the tumor proliferation (Figure 6E). No tissue damage or cardiac damage, the common side effects of Dox, were observed in the heart, liver, spleen, lungs, and kidney of the above mice (Figure 6E). Together, our data suggest that dsE-Dox is safe and effective for targeted HCC therapy.

## 4. Discussion

MSC-EVs are 30–150 nm vesicles that are secreted into the extracellular space fused with the cellular membrane [20]. Like other EVs, MSC-EVs, which contain proteins, microRNAs, mRNAs and long non-coding RNAs, are involved in intercellular communication [4]. In contrast with large living cells, nano-sized, non-living EVs would not obstruct the microvasculature, transform into inappropriate cell types, or exist as permanent grafts after the cessation of therapy [21]. The manufacturing of MSC-EVs is scalable and more suitable for process optimization as MSCs can be immortalized to ensure the standardized and reproducible production of EVs [22,23]. It has been considered as a promising tool for therapeutic purposes of immunodeficient disease and various cancers. Growing evidence has shown that MSC-EVs could be used as a vehicle to carry and deliver molecules including therapeutic genes, drugs, or RNA to the targeted cells [24]. O’Brien et al. found that MSC-derived EVs from adult human bone marrow encapsulated with miR-379 inhibited breast cancer [25]. Bagheri et al. used mouse MSC-derived EV loading with Dox as a valuable platform to significantly suppress the growth of colorectal cancer [26].

ASGPR, a liver-specific lectin, found to be a major target of B and T cell autoantigenic in patients with liver diseases, has attracted long-term attention [27]. ASGPR-mediated drug delivery [15,28] and target protein degradation [29,30] suggest that changes in glycan modification on the surface of nanoparticles could affect its interaction with recipient cells. It has been reported that neuraminidase treatment increased the EV uptake by certain cells [11] and affected EV distribution in mice [12]. Our data found that the surface of the MSC-EVs was enriched with sialic acids, and removal of the terminal linked α2–3 and α2–6 sialic acids by exogenous neuraminidase treatment could expose more Gal and GalNAc residues on the surface of the MSC-EVs and enhance its endocytosis by the recipient cells. According to these observations, an ASGPR targeting nanoparticle delivery system based on MSC-EVs loaded with Dox was designed. By examining the drug release, cellular uptake and cell cytotoxicity against HepG2 cells in vitro and in vivo, our results demonstrated that this drug delivery system could not only enhance the cell cytotoxicity of Dox, but also enhance the ASGPR targeting efficiency.

More efforts are required to improve the EV-based targeted delivery. Various approaches have been applied to isolate EVs, however, it is difficult to address all of the challenges such as batch-to-batch variation, limited yield and low purity, so a better approach is urgently needed. Moreover, the efficient delivery of multiple therapeutic molecular, and multiple-antibody and receptor/ligand coatings on EVs for better targeting can both improve the therapeutic effect.

Taken together, desialylated MSC-derived EVs loaded with Dox could be a promising candidate for drug delivery targeting hepatoma cells.

## 5. Conclusions

In conclusion, we explored the effect of the surface desialylation of MSC-EVs on the cellular uptake and targeted delivery. Neuraminidase treatment significantly increased the cellular uptake of MSC-EVs by the HepG2 cells. Encapsulating the chemotherapeutic drug Dox into desialylated MSC-EVs efficiently achieved the targeted therapy for HCC in vitro and in vivo.

## Data Availability

Not applicable.

## Supplementary Materials

The following supporting information can be downloaded at: https://www.mdpi.com/article/10.3390/cells11172642/s1, Figure S1: Characterization of the drug-loaded EVs; Figure S2: Cellular uptake of free Dox; Figure S3: The glycosylation changes of EVs treated with Neu; Figure S4: Statistics for Figure 2B; Figure S5: The cell viability assay; Table S1: Efficiency of Dox loading into EVs by different drug loading methods.
