# Supplementary material for: Desialylated Mesenchymal Stem Cells-Derived Extracellular Vesicles Loaded with Doxorubicin for Targeted Inhibition of Hepatocellular Carcinoma
Source: Cells. 2022 Aug 25;11(17):2642. doi: 10.3390/cells11172642 (PMC9454677; doi:10.3390/cells11172642)
Supplement: Supplementary file 1 [file cells-11-02642-s001.zip › cells-1800127-supplementary.pdf]

1. Doxorubicin loading into MSCs-EVs.

No.	Methods	Doxorubicin loading condition	Encapsulation efficiency (EE%)	Drug loading efficiency (DL%)
**1**	Incubation	Incubation at 37℃ for 30 min	17.40%	8.00%
**2**	Ultrasound	8% amplitude, 3 cycles of 10 s on and 10 s off cooling period	34.20%	14.6%
**3**	Electroporation	Electroporation at 150 V, 1 ms, 5 pulses in 0.2 cm cuvette	12.16%	5.73%
**4**	Freeze-thaw cycles	Freezed at −80℃ for 30 min and thawed at RT thrice.	14.33%	6.69%

Table S1. Efficiency of Dox loading into EVs by different drug loading methods. Loading capacity for Dox in EVs was measured by microplate reader at an absorbance wavelength of 480 nm. The drug encapsulation efficiency (EE%) was calculated by dividing the amount of loaded drug by which of total drug. The drug loading efficiency (DL%) was calculated by dividing the amount of loaded drug by which of drug-loaded EVs.

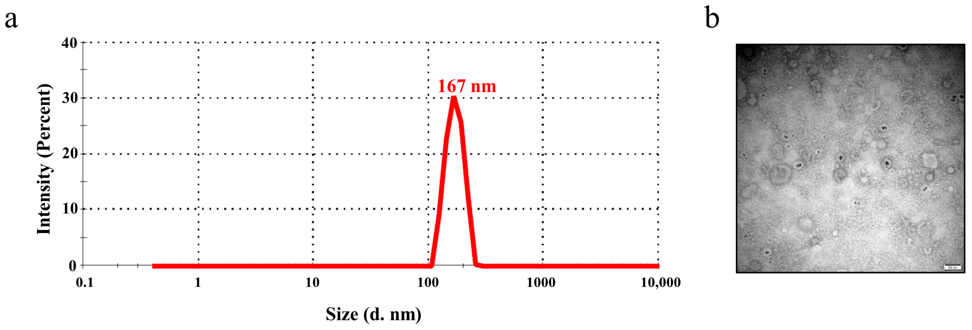

Figure S1. Characterization of drug-loaded EVs. (**a**) The size distributions of drug-loaded EVs based on intensity by DLS measurements. The peak diameters were at 167 nm. (**b**) Representative TEM images of drug-loaded EVs. Scale bar = 100 nm.

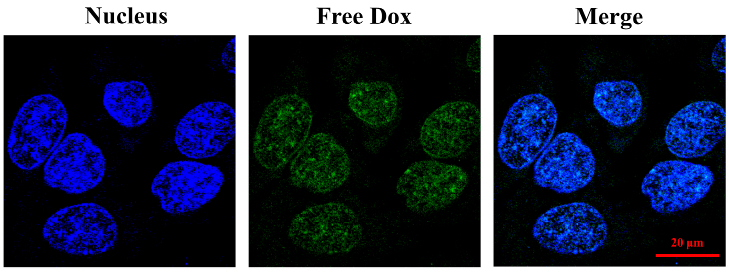

Figure S2. Cellular uptake of free Dox. HepG2 cells were incubated with free Dox (10 μmol/L) for 1 h and photographed under confocal microscopy. Blue, Hoechst 33258 for nucleus. Green, autofluorescence of Dox. Scale bar, 20 μm.

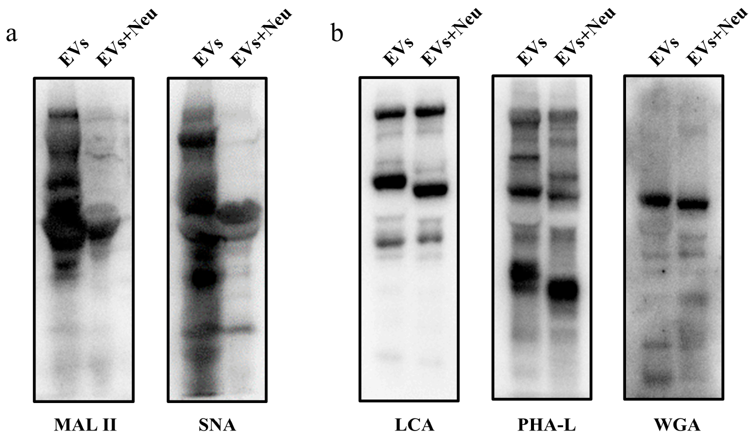

Figure S3. The glycosylation changes of EVs treated with Neu.(**a**) The level of α-2,3 and α-2,6 sialic acid of EVs were detected by lectin blotting with 4 U neuraminidase treatment at 37℃ for 30 min. (**b**) Levels of fucose, complex type N-glycans and GlcNAc on EVs was detected by lectin blotting under the same treatment conditions as (a).

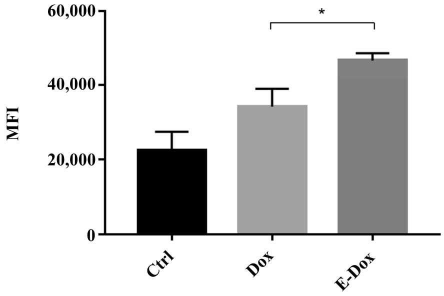

Figure S4. Statistics for Figure 2B. The data were presented as the mean values ± SD. * $p < 0.05$

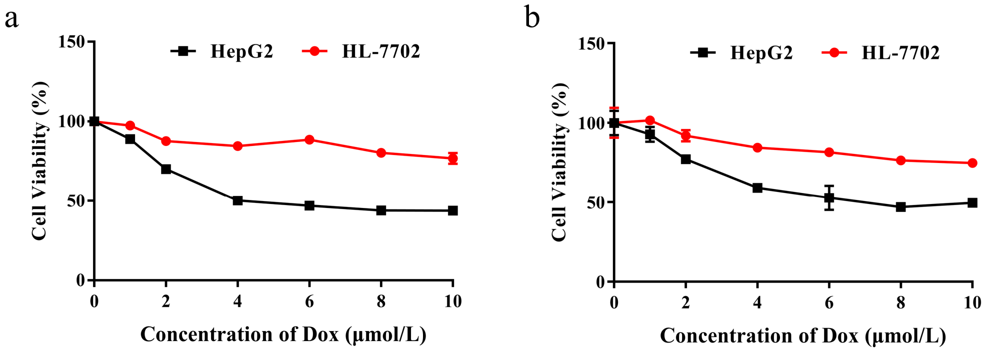

Figure S5. Cell viability assay. (**a, b**) HepG2 and HL-7702 cells were treated with desialylated Dox-loaded EVs and free Dox at different concentrations for 48 h.
